# Functional Biodegradable Nanocomposites

**DOI:** 10.3390/nano12142500

**Published:** 2022-07-21

**Authors:** Agueda Sonseca, Coro Echeverría, Daniel López

**Affiliations:** 1 MacroEng Group, Instituto de Ciencia y Tecnología de Polímeros (ICTP-CSIC), C/Juan de la Cierva 3, 28006 Madrid, Spain; 2 Interdisciplinary Plataform for “Sustainable Plastics towards a Circular Economy” (SUSPLAST-CSIC), Madrid, Spain

Over 367 million tons of plastics are produced annually worldwide, and the growth of plastic pollution has become a global concern [1]. Environmental issues related to the persistence of plastic waste have urged the development of more sustainable biodegradable alternatives. Consequently, in 2018, the European Commission adopted a circular economy plan for the management of plastics based on innovative research on polymers derived from natural resources [2]. Thus, biodegradable polymers have been effectively developed over the last few years as promising alternatives to mostly non-degradable commodity polymers, meeting the demands of a broad range of fields, including the medical, packaging, agricultural, personal care, and automotive industries [3].

Individually, biodegradable polymers do not possess physical properties or mechanical strengths comparable to their non-degradable counterparts, limiting their application. Significant research efforts have been made for the development of biodegradable polymeric formulations with mechanical and physical properties comparable to those of non-biodegradable ones [4]. As a result, biodegradable nanocomposites entered the research scene, offering the possibility of new, enhanced properties and fields of application [5].

One of the reviews in this Special Issue focuses on the application of biodegradable and biocompatible nanocomposites in electronics, highlighting the need for degradable functional systems based on nanocomposites to deal with the problem of electronic waste [6]. Nanoparticles have also found applications in nanomedicine, providing unique properties and great advantages thanks to their small size that is favorable from a therapeutic point of view. However, their safety has been questioned many times. In this context, biodegradable nanomaterials, degradable under biological conditions, hold great promise in the biomedical field, and the latest advances are reviewed in this Special Issue by Su et al. [7].

The properties of nanocomposites depend not only on the properties of individual materials, but also on their interfacial interactions and morphology, which are significantly affected by processing methods. In this context, Echeverría et al. present a detailed rheological study that investigates how gold nanoparticles (AuNP) affect the properties of a hybrid poly(acrylamide-co-acrylic acid) P(AAm-co-AAc) microgel matrix. The knowledge presented through this work facilitates the prediction of system behavior, consequently allowing the preparation of reproducible systems, for instance, as injectable systems [8]. Bardot et al. review the development of nanocomposites based on polylactic acid (PLA), a biodegradable biopolymer obtained from agricultural products, by means of fused deposition modelling (3D printing). They demonstrate the possibility of obtaining biodegradable systems without compromising mechanical robustness, which is key in industrial applications [9].

As evidenced in the review described above, polylactic acid (PLA) represents a promising alternative to mostly non-degradable commodity polymers; moreover, the modulation of its mechanical performance can be controlled with nanocomposites formation and specific processing methods. Messin et al. developed multi-nanolayered nanocomposites via the coextrusion of polylactic acid and poly(butylene succinate-*co*-butylene adipate) filled with nanoclays in order to obtain enhanced water barrier properties [10]. Sonseca et al. developed plasticized PLA nanocomposites with potential application for use as antibacterial food packaging degradable materials, incorporating silver nanoparticles obtained from a green synthesis procedure. The same materials were demonstrated to be useful as shape memory nanocomposites for potential medical application, thanks to the synergistic effect of lactic acid oligomer (OLA) and silver nanoparticles. The incorporation of OLA as a plasticizer located the glass transition of the system near to the physiological one, while the silver nanoparticles fastened the recovery process and imparted antimicrobial activity [11,12]. Nazmul et al. produced scalable environmentally friendly smart interactive textiles by means of melt spun thermoplastic conductive yarns based on PLA, polypropylene (PP), and their mixtures (PLA/PP) [13].

In summary, this Special Issue presents several examples of the latest advances in functional biodegradable nanocomposites for different applications. We would like to thank all authors for contributing to this collection, and we hope readers will find the content interesting, enjoyable, and useful.
